# Dosimetric evaluation of LINAC-based single-isocenter multi-target multi-fraction stereotactic radiosurgery with more than 20 targets: comparing MME, HyperArc, and RapidArc

**DOI:** 10.1186/s13014-024-02416-7

**Published:** 2024-02-07

**Authors:** Hyunuk Jung, Jihyung Yoon, Olga Dona Lemus, Sean Tanny, Yuwei Zhou, Michael Milano, Kenneth Usuki, Sara Hardy, Dandan Zheng

**Affiliations:** Department of Radiation Oncology, University of Rochester, Rochester, NY USA

**Keywords:** Brain metastases, Stereotactic radiosurgery, LINAC-based SRS, HyperArc, RapidArc, MME

## Abstract

**Background:**

To compare the dosimetric quality of three widely used techniques for LINAC-based single-isocenter multi-target multi-fraction stereotactic radiosurgery (fSRS) with more than 20 targets: dynamic conformal arc (DCA) in BrainLAB Multiple Metastases Elements (MME) module and volumetric modulated arc therapy (VMAT) using RapidArc (RA) and HyperArc (HA) in Varian Eclipse.

**Methods:**

Ten patients who received single-isocenter fSRS with 20–37 targets were retrospectively replanned using MME, RA, and HA. Various dosimetric parameters, such as conformity index (CI), Paddick CI, gradient index (GI), normal brain dose exposures, maximum organ-at-risk (OAR) doses, and beam-on times were extracted and compared among the three techniques. Wilcoxon signed-rank test was used for statistical analysis.

**Results:**

All plans achieved the prescribed dose coverage goal of at least 95% of the planning target volume (PTV). HA plans showed superior conformity compared to RA and MME plans. MME plans showed superior GI compared to RA and HA plans. RA plans resulted in significantly higher low and intermediate dose exposure to normal brain compared to HA and MME plans, especially for lower doses of ≥ 8Gy and ≥ 5Gy. No significant differences were observed in the maximum dose to OARs among the three techniques. The beam-on time of MME plans was about two times longer than RA and HA plans.

**Conclusions:**

HA plans achieved the best conformity, while MME plans achieved the best dose fall-off for LINAC-based single-isocenter multi-target multi-fraction SRS with more than 20 targets. The choice of the optimal technique should consider the trade-offs between dosimetric quality, beam-on time, and planning effort.

## Introduction

Brain metastases represent a significant and prevalent condition, affecting approximately 10–40% of cancer patients [1]. To address this challenge, stereotactic radiosurgery (SRS), fractionated SRS (fSRS), and whole brain radiotherapy (WBRT) with a simultaneous integrated boost (SIB) have emerged as a promising non-invasive treatment modality [2–4]. SRS involves the precise and accurate delivery of a high dose of radiation to patients with one or a few brain metastases (generally less than 4) in a single session, while fSRS, on the other hand, spreads the radiation dose over multiple treatment sessions, generally 3–5 fractions, for patients with even more metastases. This fractionated approach allows for the delivery of effective doses to the multiple-target while minimizing damage to surrounding healthy brain tissue [5, 6]. Various techniques, including the use of conventional linear accelerator (LINAC), Gamma Knife (Elekta AB, Stockholm, Sweden), and CyberKnife (Accuray, Sunnyvale, CA), have been employed for SRS/fSRS [7–10].

Among these techniques, LINAC-based SRS/fSRS offers several advantages [10]. Firstly, it is widely accessible, making it a practical option for many healthcare facilities. Additionally, LINAC-based SRS/fSRS allows for the treatment of multiple brain metastases using a single isocenter. This approach not only reduces treatment time but also mitigates the risk of setup errors [11]. However, LINAC-based SRS/fSRS poses certain challenges that need to be addressed to optimize its effectiveness.

One such challenge is the necessity for accurate and efficient treatment planning. Given the complex nature of multiple brain metastases, an in-depth understanding of the dosimetric considerations is crucial to ensure optimal radiation delivery [11]. This involves the modulation of beam intensity, shape, and direction to generate treatment plans that achieve superior dose distribution and conformity. Moreover, minimizing the irradiation of normal brain tissue is of paramount importance to preserve neurological function and minimize potential side effects [12, 13].

In recent years, different techniques have been developed to enhance the capabilities of LINAC-based SRS/fSRS in treating multiple brain metastases with a single isocenter. Examples include the dynamic conformal arc (DCA) technique in the BrainLAB Multiple Metastases Element (MME) module (BrainLAB, Munich, Germany) [14, 15], the HyperArc technique (Varian Medical Systems, Palo Alto, CA), and the general volumetric modulated arc therapy (VMAT) technique such as using RapidArc in Varian Eclipse (Varian Medical Systems, Palo Alto, CA) [16–20]. These techniques aim to optimize the dosimetric performance and clinical outcomes of LINAC-based SRS/fSRS, particularly for cases involving multiple brain metastases. However, there is a dearth of comprehensive comparative studies evaluating the efficacy and dosimetric quality of these systems, especially for treatments with large numbers of targets.

Therefore, the objective of this research paper is to evaluate and compare the dosimetric quality of MME, HyperArc (HA), and RapidArc (RA) for LINAC-based single isocenter multiple brain metastases fSRS involving more than 20 targets. By conducting a comprehensive analysis, this study aims to address the gaps in current knowledge and contribute to the existing body of knowledge regarding treatment planning and optimization strategies for LINAC-based fSRS. The findings of this research will provide valuable insights into the selection and utilization of the most effective technique for managing large numbers of brain metastases in a clinical setting.

## Methods

Approved by our Institutional Human Subjects Review Board, in this study, ten patients who received single-isocenter fSRS between June 2020 and May 2022 in our department were selected. The patient characteristics and treatment parameters are summarized in Table 1. The number of metastases per patient ranged from 20 to 37, for a total of 263 metastases. Minimum, maximum, and median PTV volumes are 0.02 cc, 19.64 cc, and 0.22 cc, respectively. All patients were treated with frameless LINAC-based single-isocenter fSRS using a commercial stereotactic mask system (BrainLAB, Munich, Germany). Prescribed doses (Rx) for these patients included 21 Gy in 3 fractions, 24 Gy in 3 fractions, 25 Gy in 5 fractions, and 27 Gy in 3 fractions. All plans were optimized to deliver the prescription dose to at least 95% of PTV (see description below), allowing a maximum dose of less than 150% of the prescription dose. The dose constraints for organs-at-risk (OARs) followed the guidelines in AAPM TG101 and HyTEC [21–23]. All treatments were performed with 6MV flattening-filter-free beams on a Varian Edge equipped with HD120 MLC (Varian, CA, USA), using oblique kV-kV imaging guidance with the ExacTrac system (BrainLAB, Munich, Germany) for patient setup and position verification at all couch angles.

**Table 1:** Summary of patient characteristics and treatment parameters

Parameter	Value
Number of patients	10
Median age	69.5
Total number of PTVs	263
Number of PTVs per patient	
Median	24.5
Range	20–37
PTV volume [cc]	
Median	0.22
Range	0.02–19.64
Prescribed Dose	# of targets
21Gy in 3 fractions	62
24Gy in 3 fractions	136
25Gy in 5 fractions	20
27Gy in 3 fraction	45

### Planning preparation

BrainLAB Elements was utilized for image registration between computed tomography (CT) and magnetic resonance imaging (MRI), distortion correction for MRI, and delineation of targets and OARs. For CT simulation, a slice thickness of 1.25 mm and an axial resolution of 0.8 mm were used. For MRI, a slice thickness of 1 mm and an axial resolution of about 1 mm was used. In Elements, deformable registration was utilized for image registration and MRI distortion correction, and OARs were contoured by using the anatomy mapping function. GTVs were contoured on MRI fused to the treatment planning CT by the attending physician and PTVs were generated by adding a 0.5 mm to 1.5 mm margin to the GTV contour to incorporate setup uncertainties. The size of the margin was determined by the physician’s discretion considering the size and location of the target.

For each patient, a treatment plan was generated using each of the three SRS planning techniques: Eclipse-RA, Eclipse-HA, and Elements-MME. The RA and HA plans were generated in Eclipse after importing the images and contours into Eclipse. Of note, eight patients were treated clinically with MME plans and two were treated with RA plans. All other plans were retrospectively generated for comparison in this study, by two experienced SRS planners. All plans in this study were generated following our institutional planning standards, and the two planners performed cross-checks of each other’s plans to minimize inter-planner variability.

### MME

MME plans were created using BrainLAB Elements MME version 3.0 which is a dedicated treatment planning system for multiple brain metastases and offers a highly automated planning workflow. MME automatically places the isocenter at the geometric center of mass of all target volumes. All MME plans used 7 non-coplanar DCA beams at different couch angles. The gantry angles of each arc are initially set to default values of 10–170 with a couch angle ranging from 0 to 90, and 190–350 with a couch angle ranging from 270 to 360, and then automatically adjusted during optimization. In addition to arc angle/length, other beam parameters are also automatically adjusted by the MME system, including aperture opening, collimator angle, couch angle, and beam weighting, to attain the prescribed dose for every target with the highest conformity and the OAR dose goals. In addition, two partial arcs (clockwise and counter-clockwise) were maximally used per couch angle to treat all metastases. All MME plans were calculated using the system’s Monte Carlo algorithm with a dose grid size equaling the CT resolution.

### RapidArc (RA) and HyperArc (HA)

RapidArc (RA, Varian Medical Systems, Palo Alto, CA) and HyperArc (HA, Varian Medical Systems, Palo Alto, CA) both utilized the VMAT technique. For the RA plans, a pre-defined template with seven different couch angles was used for all patients which included 70, 40, 20, 0, 340, 310, and 280 degrees. Collimator angles were manually optimized to minimize island blocking which tends to increase the MLC pattern complexity and the normal brain exposure. To avoid collision, the range of gantry angles was chosen at 20–170 for couch angles 70°, 40°, and 20°, and 190–340 for couch angles 340°, 310°, and 280°. For the field with 0° couch, a 120–240 CCW arc was used with an avoidance sector in 30–330 to prevent excessive doses to optical structures such as eyes, lenses, optic nerves, chiasm, etc. For the HA plans, a templated treatment field arrangement was used consisting of four arcs: one 360-degree full arc at couch 0, and three 180-degree half arcs with a couch kick of 45, 315, and 270 degrees, respectively. Collimator angles were automatically optimized and determined during a plan preparation process based on geometric information of PTVs. For both RA and HA plans, the dose was calculated using the AcurosXB algorithm (version 15.6.06) with a 1 mm dose grid size.

### Plan comparison and data evaluation

Plan evaluation was performed in Eclipse for all plans, with the MME plans imported into Eclipse for evaluation. Quantitative analysis was conducted using ClearCheck (Radformation, NY), extracting several dosimetric parameters from the dose-volume histograms for all PTVs, OARs, and normal brain tissues across all treatment plans as described below.

RTOG conformity index (CI), Paddick CI, and Gradient index (GI) are well-known indices to analyze how tightly the prescription dose is conforming to the target and how steep the dose fall-off is around the target [24, 25]. The Paddick CI also accounts for target coverage.

CI is defined as CI = TV/PTV where TV is the treated volume enclosed by the 100% prescription isodose surface and PTV is the planning target volume.

Paddick CI is defined as Paddick CI = (TVPIV)^2^/(TV*PIV), where TVPIV is the target volume covered by the prescription isodose volume, TV is the target volume or PTV, and PIV is the prescription isodose volume.

GI is defined as GI = PIVhalf/PIV, where PIVhalf is the prescription isodose volume at half of the prescription isodose and PIV is the prescription isodose volume. In this study, the typical GI definition was not applicable for some targets due to the larger number of metastases and the bridged isodose distributions for targets close to each other. To mitigate the impact of this, we generated a region of interest (ROI) around each PTV to calculate the PIVhalf and PIV within the ROI. ROI margins of 3, 5, and 10 mm from the PTVs were applied and tested.

We also evaluated the volume of normal brain (brain minus GTV) receiving doses of ≥ 23, 18, 12, 8, and 5 Gy, respectively. Moreover, the maximum point dose (dose to 0.035cc of the structure) to the brainstem, chiasm, and left/right optic nerve was evaluated. Lastly, the total beam-on time for each plan was also extracted for comparison.

### Statistics

To statistically evaluate the extracted dosimetric parameters across the three techniques, a Wilcoxon signed-rank test was used to compare each matched pair of the three techniques. The difference was considered statistically significant if *p* < 0.05.

## Results

**Fig. 1:**
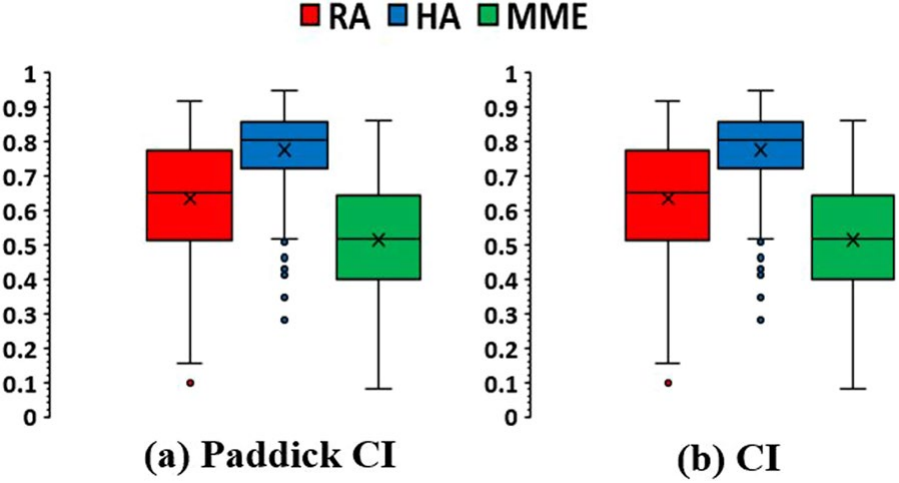
Comparison of Paddick conformity index (Paddick CI) and conformity index (CI) for the three different planning techniques

All plans created in this study were clinically acceptable and achieved the prescribed dose coverage goal which is at least 95% of PTV receiving the prescription dose. The graphical comparison presented in Fig. 1 illustrates the two different types of conformity indices across the three fSRS plans. With both conformity indices, the analysis reveals that HA plans exhibit superior conformity compared to RA and MME plans. Median Paddick CI was 0.803 for HA, compared with 0.651 for RA and 0.517 for MME (*p* < 0.05 for both); median CI was 1.195 for HA, compared with 1.432 for RA and 1.86 for MME (*p* < 0.05 for both).

**Fig. 2:**
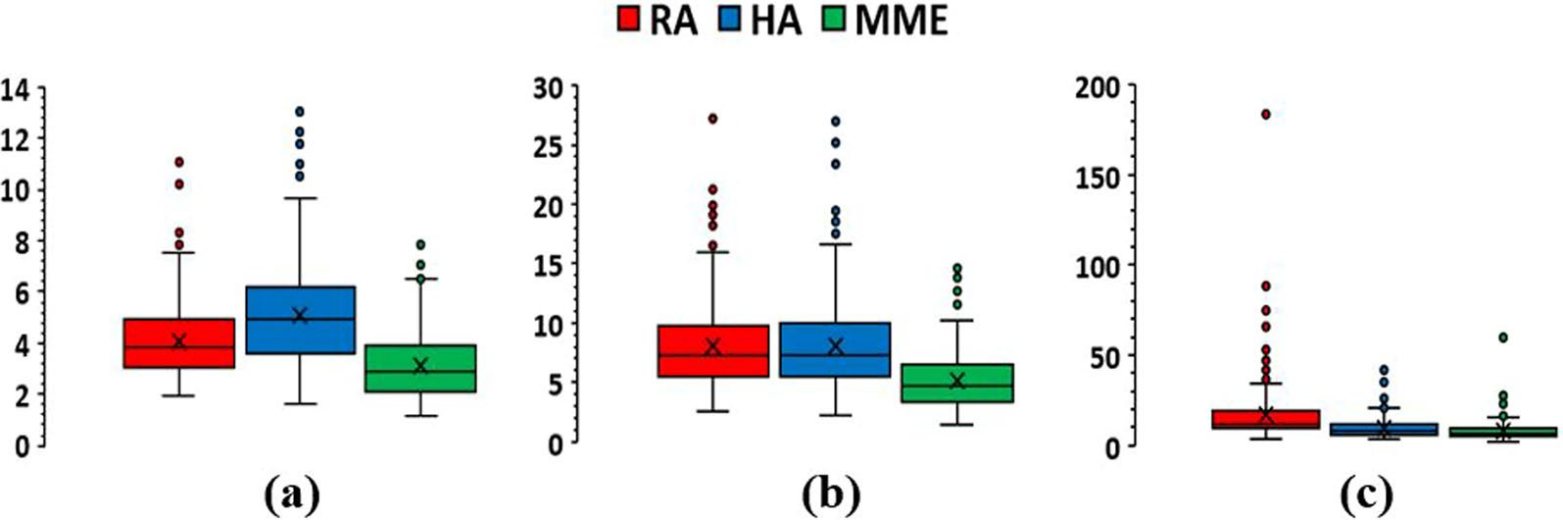
Comparison of Gradient index results with three different margins around the target. **a** GI with a 3 mm margin, **b** GI with a 5 mm margin, and **c** GI with a 10 mm margin

Figure 2 provides a graphical representation of the results for GI comparing the three techniques. Results using the three different ROI margins from the PTV are separately plotted. As expected, GI values for some cases increase with the ROI margin. But regardless of the ROI margin, MME plans tend to exhibit superior GI compared with RA and HA plans, with a median GI of 2.89, 4.69, and 6.67 for 3 mm, 5 mm, and 10 mm ROI margins, respectively. The superior GI of MME plans from the 3 mm and 5 mm margin ROI calculations was significant compared with RA and HA plans (*p* < 0.05 for both). In contrast, from the largest 10 mm margin ROI calculation, MME and HA plans showed similar GIs, while RA plans showed slightly inferior GI (*p* < 0.05). From the smallest 3 mm margin ROI calculation, besides MME resulting in the best GI, RA also achieved better GI than HA (*p* < 0.05).

**Fig. 3:**
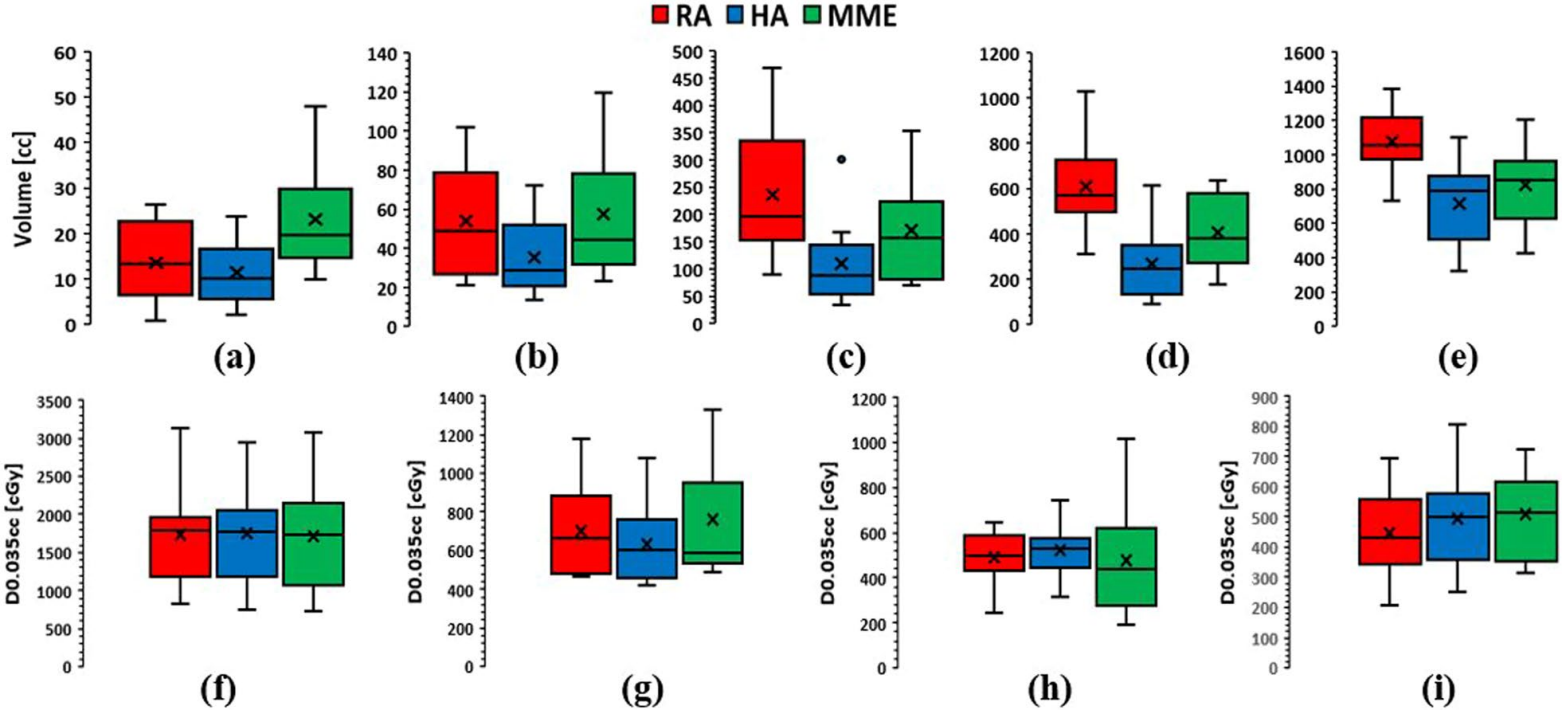
Comparison of the volume of normal brain receiving **a** 23Gy, **b** 18Gy, **c** 12Gy, **d** 8Gy, and **e** 5Gy and the maximum dose to **f** brainstem, **g** chiasm, **h** left optic nerve, and **i** right optic nerve for three different planning techniques

Figure 3a–e displays the box plots for comparison of the volume of normal brain tissue receiving ≥ 23Gy, 18Gy, 12Gy, 8Gy, and 5Gy. Overall, the HA plans achieved the lowest normal brain dose across all assessed dose volume points. In addition, the RA plans resulted in significantly higher low and intermediate dose exposure to the normal brain compared to HA and MME plans, especially for lower doses i.e. 8 Gy and 5 Gy. For V23Gy, MME was higher than HA and RA. In the case of the maximum dose to other OARs, i.e. brainstem, chiasm, and optic nerves, no general trends can be found in this study and the three techniques performed similarly as shown in Fig. 3f–i. All the different dosimetric parameters are summarized in Table 2.

**Table 2:** Different dosimetric parameters (i.e., Paddick CI, CI, GI with different margins, and normal brain volumes [cc] receiving ≥ 23Gy, 18Gy, 12Gy, 8Gy, and 5Gy for the different treatment techniques). All values indicate Median ± Standard deviation

	PCI	CI	GI_3	GI_5	GI_10	V23Gy	V18Gy	V12Gy	V8Gy	V5Gy
RA	0.65 ± 0.17	1.43 ± 0.83	3.85 ± 1.41	7.32 ± 3.77	12.05 ± 16.44	13.6 ± 8.22	49.25 ± 26.9	197.6 ± 114.79	571.65 ± 188.45	1055.9 ± 173.03
HA	0.8 ± 0.12	1.2 ± 0.33	4.93 ± 1.96	7.33 ± 3.87	7.98 ± 5.88	10.3 ± 7.07	29.1 ± 18.72	88.2 ± 75.21	249.1 ± 154.72	793.7 ± 237.9
MME	0.52 ± 0.16	1.86 ± 1.31	2.89 ± 1.3	4.69 ± 2.43	6.67 ± 5.74	19.85 ± 10.93	44.6 ± 32.35	157.25 ± 93.52	381 ± 155.9	856.95 ± 215.73

To further illustrate the dosimetric parameter comparisons between each pair of techniques, the median differences between each pair are shown in Table 3, where the values indicate the result of subtracting the row-listed technique from the column-listed technique. Most of the differences were statistically significant (*p* < 0.05) from the Wilcoxon signed-rank test, except for a few cases with *p* ≥ 0.05 indicated in bold.

**Table 3:** Median differences for every matched pair plan comparison among three planning techniques

	Metric	HA	MME
**RA**	Paddick CI	− 0.15	0.13
	CI	0.24	− 0.43
	GI_3mm	− 1.08	0.96
	GI_5mm	− 0.01 **(*p* = 0.103)**	2.63
	GI_10mm	4.08	5.38
	V23Gy [cc]	3.3 **(*p* = 0.084)**	− 6.25
	V18Gy [cc]	20.15	4.65 **(*p* = 0.92)**
	V12Gy [cc]	109.4	40.35 **(*p* = 0.084)**
	V8Gy [cc]	322.55	190.65
	V5Gy [cc]	262.2	198.95
**HA**	Paddick CI	N/A	0.29
	CI		− 0.67
	GI_3mm		2.04
	GI_5mm		2.64
	GI_10mm		1.3
	V23Gy [cc]		− 9.55
	V18Gy [cc]		− 15.5
	V12Gy [cc]		− 69.05
	V8Gy [cc]		− 131.9
	V5Gy [cc]		− 63.25 **(*p* = 0.065)**

**Fig. 4:**
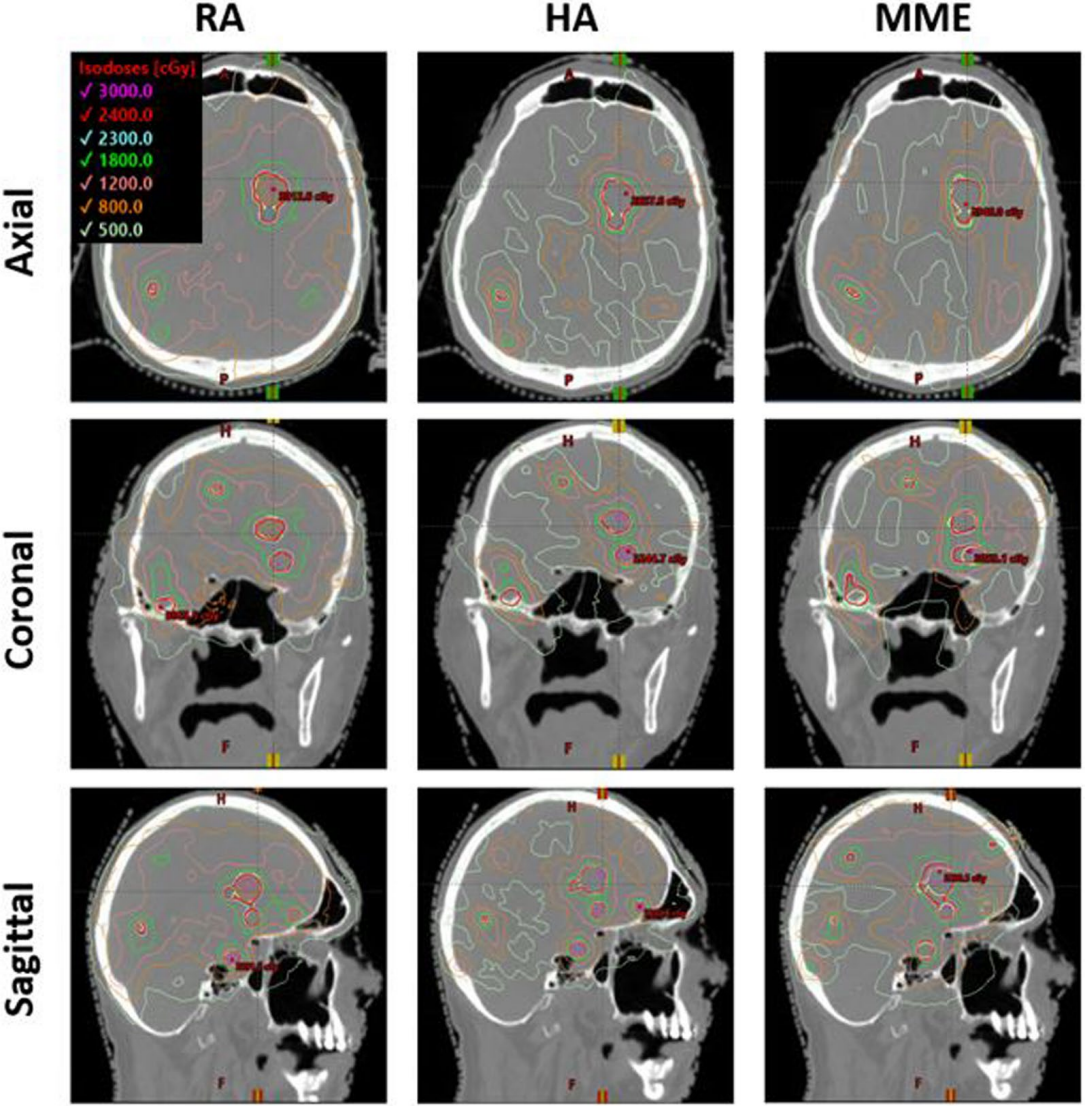
Comparison in dose distribution of case#2 which has 25 PTVs and the Rx is 24Gy to 95% of PTVs. Yellow contours indicate PTVs

As a visual comparison of the dosimetric results, Fig. 4 shows an example dose distribution of case#2 which has a total of 25 PTVs. The axial, coronal, and sagittal views showcase both isolated PTVs and closely neighboring PTVs. Overall, the HA plan appears to have superior high-dose target coverage and conformity even to closely-neighboring targets and superior low-dose spread.

Lastly, we compared the total beam-on times listed in Table 4 which are approximated based on total monitor units divided by a 1400 MU/min dose rate. Overall, RA and HA plans had similar beam-on times. In contrast, the beam-on time of MME plans was about two times longer than other plans.

**Table 4:** Comparison of the total beam-on time in minutes per patient with 1,400 MU/min dose rate delivery

Beam-on time				
Patient#	# of target	RA	HA	MME
1	27	4.13	4.47	8.05
2	25	3.06	3.16	8.40
3	36	3.29	3.03	6.86
4	24	2.57	2.17	7.67
5	24	3.86	5.71	6.99
6	37	3.12	2.72	8.93
7	20	2.13	1.79	4.13
8	27	2.97	3.44	7.56
9	22	3.59	3.67	6.63
10	21	2.31	8.39	2.67
Mean	26.3	3.10 ± 0.61	3.28 ± 1.08	7.36 ± 1.29

## Discussion

In this study, we evaluated and compared the dosimetric quality of MME, HyperArc and, RapidArc for LINAC-based single isocenter, multiple brain metastases multi-fraction SRS involving more than 20 targets. By conducting a comprehensive analysis, we aimed to address the gaps in current knowledge and contribute to the existing body of knowledge regarding treatment planning and optimization strategies for LINAC-based fSRS. The findings of this research provide valuable insights into the selection and utilization of the most effective technique for managing large numbers of brain metastases in a clinical setting.

*Dose and planning effort with increasing number of targets* Previous studies have shown that the number of targets, their size, shape, and location, as well as the number of arcs, total monitor units, and beam modulation, can affect the dosimetric outcomes and treatment efficiency of LINAC-based fSRS [9, 14, 16, 18, 19]. However, most of these studies focused on cases with fewer than 20 targets, mostly fewer than 10 targets, and there is a paucity of investigation on the dosimetric challenges and trade-offs for cases with large numbers of targets. Our study fills this gap by comparing three different techniques for such complex cases and demonstrates the importance of choosing the appropriate technique for achieving optimal plan quality and delivery time. We found that MME plans had the longest beam-on time, followed by HA and RA, which were comparable. The longer delivery time for MME plans is mainly due to the use of two partial arcs per couch angle to treat all targets in the forward fashion with DCA, which increases the number of beam segments and monitor units. On the other hand, HA and RA plans use fewer arcs with higher modulation to achieve similar or better target coverage and conformity. However, in our experience, the planning effort for MME plans is generally lower than for HA and RA plans, as MME is a highly automated planning system that streamlines image fusion, MRI distortion correction, and OAR contouring, and adjusts the beam parameters automatically, while HA and RA plans require more preparation time for planning, such as image fusion and OAR contouring, and more inputs for the optimization. Therefore, there is a trade-off between planning effort and delivery time for the three techniques, and the choice of technique may depend on the availability of planning resources and treatment slots.

*Strengths and weaknesses of the three techniques* Our study showed that HA plans achieved the best target conformity, and MME plans are often least conformal. This may be attributed to the HA-specific optimization algorithm, which automatically adjusts the collimator angles, arc lengths, and MLC apertures with VMAT to minimize the dose spillage and interplay effects. As for MME, achieving high conformity is increasingly challenging with forward DCA with the increasing number of metastases and hence planning complexity. RA plans had slightly lower conformity and similar normal brain doses as HA plans in the higher dose region, but significantly higher doses in the low dose region. This may be due to the use of seven non-coplanar arcs compared with HA and larger MLC and jaw openings compared with MME, which increases the dose to the surrounding tissues, especially for lower doses. MME plans achieved the best gradient index, followed by HA and RA plans. The better gradient index of MME plans can be explained by the use of dynamic conformal arcs that match the beam aperture to the target projection, which may result in steeper dose fall-off. However, MME plans also had the lowest conformity and the highest volume of normal brain receiving ≥ 23 Gy, which may increase the risk of radionecrosis [21, 22]. This may be due to the challenges of forward planning with DCA in handling these more complicated cases with higher numbers of targets, sometimes leaving dose bridges between nearby targets. In addition, MME plans had the longest beam-on time, which may increase the risk of intra-fraction motion and patient discomfort. Therefore, each technique has its strengths and weaknesses, and the choice of technique may depend on the dosimetric goals and clinical constraints for each case.

*Comparison with published literature* Since our study was focused on the high-number target cases, it was hard to directly compare the trends with already published studies. Raza et al. [20] compared Elements MME with Eclipse HA plans in 36 patients with a median number of 9 (2–25) brain metastases. They observed that MME plans showed favorable Paddick CI (median Paddick CI, 0.75 vs 0.65) and lower V12Gy (median V12Gy, 13.65 vs 18.51 cm^3^) compared with HA plans, which were different from our results. This is possibly due to the differences in the number and size of lesions in the two cohorts and, in addition, the different dose calculation algorithms in the two studies. In their study, the pencil beam algorithm and the anisotropic analytical algorithm (AAA) were used for MME and HA plan calculation, respectively, which was less accurate than the algorithms used in our study. Vergalasov et al. [26] conducted a multi-institutional dosimetric comparison using four different treatment planning options for SRS with multiple brain metastases, HA, RA, MME, and GammaKnife, on 16 patients with a range of 4–10 metastases each, for a total of 112 brain metastases. In agreement with our results, they found that HA plans resulted in superior CI when compared with GammaKnife and MME plans, and HA plan had a lower volume of normal brain dose endpoints than MME which is consistent with our results. However, due to the differences in the number and location of the targets, the results and trends of these studies may not be directly comparable, and additional studies are needed to further validate the findings.

*Challenges of GI calculation for high-number target cases* The gradient index is a commonly used dosimetric index to evaluate the dose fall-off around the target. However, the calculation of GI for high-number target cases is challenging, as the conventional definition of GI may not be applicable for cases with closely spaced or overlapping targets. In such cases, the prescription isodose volume at half of the prescription dose (PIVhalf) may not be well-defined, as it may include the dose from adjacent targets. Therefore, alternative methods need to be identified to calculate GI for high-number target cases. In our study, we used a region of interest (ROI) around each target in lieu of body to confine the dose volumes to mitigate this. However, there is still a delicate interplay in selecting the appropriate margin to create the ROI. If the margin is too small, it may not completely encompass the PIVhalf, therefore underestimating GI; but if it is too large, it may encroach on the dose cloud of a nearby target, therefore overestimating GI. In our study, we used the ROI method and experimented with three different margins (3, 5, and 10 mm) from the PTVs. Not surprisingly, we found that the GI values for some cases increased with the ROI margin. This may be due to the inclusion of more dose from neighboring targets with larger ROI margins, which may inflate the PIVhalf and hence increase the GI. On the other hand, with smaller ROI margins, GI could be underestimated for some targets too if the true PIVhalf spills beyond the ROI. In our study, regardless of the ROI margin in our analyses, MME plans resulted in better GI than HA and RA plans, which indicates the superiority of the MME technique in terms of dose fall-off. Nevertheless, the choice of GI calculation method and ROI margin may affect the results and comparison of GI for high-number target cases, and a standardized method is needed to ensure consistency and accuracy and to support the plan evaluation of these complex cases with high numbers of multiple metastases.

## Conclusion

This study evaluated and compared the dosimetric quality of three LINAC-based fSRS techniques for treating high-number (> 20) multiple brain metastases with a single isocenter: MME, HyperArc, and RapidArc. The results showed that HA plans achieved superior conformity and lower normal brain dose than RA and MME plans, while MME plans achieved a superior gradient index than RA and HA plans. However, further studies are needed to validate the results and to evaluate the clinical outcomes and toxicities of these techniques. Moreover, the selection of the most appropriate technique should also consider other factors, such as planning efficiency, delivery time, and patient-specific characteristics.

## Data Availability

All date generated or analyzed during this study are included in this published article.

## References

[1] Achrol AS, Rennert RC, Anders C, Soffietti R, Ahluwalia MS, Nayak L, Peters S, Arvold ND, Harsh GR, Steeg PS (2019). Brain metastases. Nat Rev Dis Primers.

[2] Churilla TM, Ballman KV, Brown PD, Twohy EL, Jaeckle K, Farace E, Cerhan JH, Anderson SK, Carrero XW, Garces YI (2017). Stereotactic radiosurgery with or without whole-brain radiation therapy for limited brain metastases: a secondary analysis of the north central cancer treatment group N0574 (Alliance) randomized controlled trial. Int J Radiat Oncol Biol Phys.

[3] Blomain ES, Kim H, Garg S, Bhamidipati D, Guo J, Kalchman I, McAna J, Shi W (2018). Stereotactic radiosurgery practice patterns for brain metastases in the United States: a national survey. J Radiat Oncol.

[4] GiajLevra N, Sicignano G, Fiorentino A, Fersino S, Ricchetti F, Mazzola R, Naccarato S, Ruggieri R, Alongi F (2016). Whole brain radiotherapy with hippocampal avoidance and simultaneous integrated boost for brain metastases: a dosimetric volumetric-modulated arc therapy study. Radiol Med.

[5] Chang EL, Wefel JS, Hess KR, Allen PK, Lang FF, Kornguth DG, Arbuckle RB, Swint JM, Shiu AS, Maor MH (2009). Neurocognition in patients with brain metastases treated with radiosurgery or radiosurgery plus whole-brain irradiation: a randomised controlled trial. Lancet Oncol.

[6] Yamamoto M, Serizawa T, Shuto T, Akabane A, Higuchi Y, Kawagishi J, Yamanaka K, Sato Y, Jokura H, Yomo S (2014). Stereotactic radiosurgery for patients with multiple brain metastases (JLGK0901): a multi-institutional prospective observational study. Lancet Oncol.

[7] Yu X, Wang Y, Yuan Z, Yu H, Song Y, Zhao L, Wang P (2020). Benefit of dosimetry distribution for patients with multiple brain metastases from non-small cell lung cancer by a Cyberknife stereotactic radiosurgery (SRS) system. BMC Cancer.

[8] Potrebko PS, Keller A, All S, Sejpal S, Pepe J, Saigal K, Kandula S, Sensakovic WF, Shridhar R, Poleszczuk J (2018). GammaKnife versus VMAT radiosurgery plan quality for many brain metastases. J Appl Clin Med Phys.

[9] Meeks SL, Mercado CE, Popple RA, Agazaryan N, Kaprealian T, Fiveash JB, Tenn S (2022). Practical considerations for single isocenter linac radiosurgery of multiple brain metastases. Pract Radiat Oncol.

[10] Skourou C, Hickey D, Rock L, Houston P, Sturt P, Faul C, Paddick I (2021). Treatment of multiple intracranial metastases in radiation oncology: a contemporary review of available technologies. BJR Open.

[11] Shaw E, Scott C, Souhami L, Dinapoli R, Kline R, Loeffler J, Farnan N (2000). Single dose radiosurgical treatment of recurrent previously irradiated primary brain tumors and brain metastases: final report of RTOG protocol 90–05. Int J Radiat Oncol Biol Phys.

[12] Blonigen BJ, Steinmetz RD, Levin L, Lamba MA, Warnick RE, Breneman JC (2010). Irradiated volume as a predictor of brain radionecrosis after linear accelerator stereotactic radiosurgery. Int J Radiat Oncol Biol Phys.

[13] Minniti G, Clarke E, Lanzetta G, Osti MF, Trasimeni G, Bozzao A, Romano A, Enrici RM (2011). Stereotactic radiosurgery for brain metastases: analysis of outcome and risk of brain radionecrosis. Radiat Oncol.

[14] Huang Y, Chin K, Robbins JR, Kim J, Li H, Amro H, Chetty IJ, Gordon J, Ryu S (2014). Radiosurgery of multiple brain metastases with single-isocenter dynamic conformal arcs (SIDCA). Radiother Oncol.

[15] Mori Y, Kaneda N, Hagiwara M, Ishiguchi T (2016). Dosimetric study of automatic brain metastases planning in comparison with conventional multi-isocenter dynamic conformal arc therapy and gamma knife radiosurgery for multiple brain metastases. Cureus.

[16] Alongi F, Fiorentino A, Gregucci F, Corradini S, Giaj-Levra N, Romano L, Rigo M, Ricchetti F, Beltramello A, Lunardi G (2019). First experience and clinical results using a new non-coplanar mono-isocenter technique (HyperArc) for Linac-based VMAT radiosurgery in brain metastases. J Cancer Res Clin Oncol.

[17] Ohira S, Ueda Y, Akino Y, Hashimoto M, Masaoka A, Hirata T, Miyazaki M, Koizumi M, Teshima T (2018). HyperArc VMAT planning for single and multiple brain metastases stereotactic radiosurgery: a new treatment planning approach. Radiat Oncol.

[18] Liu H, Thomas EM, Li J, Yu Y, Andrews D, Markert JM, Fiveash JB, Shi W, Popple RA (2020). Interinstitutional plan quality assessment of 2 linac-based, single-isocenter, multiple metastasis radiosurgery techniques. Adv Radiat Oncol.

[19] Hartgerink D, Swinnen A, Roberge D, Nichol A, Zygmanski P, Yin FF, Deblois F, Hurkmans C, Ong CL, Bruynzeel A (2019). LINAC based stereotactic radiosurgery for multiple brain metastases: guidance for clinical implementation. Acta Oncol.

[20] Raza GH, Capone L, Tini P, Giraffa M, Gentile P, Minniti G (2022). Single-isocenter multiple-target stereotactic radiosurgery for multiple brain metastases: dosimetric evaluation of two automated treatment planning systems. Radiat Oncol.

[21] Benedict SH, Yenice KM, Followill D, Galvin JM, Hinson W, Kavanagh B, Keall P, Lovelock M, Meeks S, Papiez L (2010). Stereotactic body radiation therapy: the report of AAPM Task Group 101. Med Phys.

[22] Milano MT, Grimm J, Niemierko A, Soltys SG, Moiseenko V, Redmond KJ, Yorke E, Sahgal A, Xue J, Mahadevan A (2021). Single- and multifraction stereotactic radiosurgery dose/volume tolerances of the brain. Int J Radiat Oncol Biol Phys.

[23] Milano MT, Grimm J, Soltys SG, Yorke E, Moiseenko V, Tome WA, Sahgal A, Xue J, Ma L, Solberg TD (2021). Single- and multi-fraction stereotactic radiosurgery dose tolerances of the optic pathways. Int J Radiat Oncol Biol Phys.

[24] Paddick I (2000). A simple scoring ratio to index the conformity of radiosurgical treatment plans. Technical note J Neurosurg.

[25] Paddick I, Lippitz B (2006). A simple dose gradient measurement tool to complement the conformity index. J Neurosurg.

[26] Vergalasova I, Liu H, Alonso-Basanta M, Dong L, Li J, Nie K, Shi W, Teo BK, Yu Y, Yue NJ (2019). Multi-institutional dosimetric evaluation of modern day stereotactic radiosurgery (SRS) treatment options for multiple brain metastases. Front Oncol.

